# Testicular Radiotherapy: A Challenging Irradiation Site

**DOI:** 10.7759/cureus.37638

**Published:** 2023-04-16

**Authors:** Zineb Dahbi, Reyzane Elmejjabar, Rim Alami, Fadila Kouhen

**Affiliations:** 1 Radiotherapy, International University Hospital Cheikh Khalifa, Mohammed VI University of Health Sciences, Casablanca, MAR; 2 Radiotherapy, Mohammed VI University of Health Sciences, Casablanca, MAR; 3 Radiation Oncology, Mohammed VI University of Health Sciences, Casablanca, MAR

**Keywords:** onco-urology, adjuvant radiotherapy, target volumes testicular radiotherapy, testis cancer, testicular radiotherapy

## Abstract

Testicular radiation therapy is a crucial component of the overall treatment of certain neoplasms. Yet, it remains challenging due to the unique anatomic location of the testicles, their specific radiation tolerance, and the lack of a standardized treatment workflow. In this article, we present the case of a 78-year-old patient with primary testicular lymphoma and describe the technical aspects of his radiation therapy. The challenge was to achieve a comfortable, reproducible, and effective treatment position while protecting the penis and covering the superficial layers of the scrotum. We used a total body restraint system and performed a second simulated CT scan with a bolus. The entire scrotum was delineated as the clinical target volume, with an additional 1 cm margin to obtain the planning target volume. This case highlights the importance of careful planning and personalized treatment approaches in testicular irradiation and underscores the need for further research and standardization in this complex irradiation site.

## Introduction

Testicular irradiation is challenging due to the complex anatomy and function of the testes, the high risk of relapse, potential side effects, and the need for precise treatment planning. The location of the testes, their sensitivity to radiation, and the risk of contralateral testis relapse require careful delivery of radiation while minimizing exposure to healthy tissues. Preservation of testicular function, management of potential side effects, and coordination with other treatments are also important considerations. Despite these challenges, testicular irradiation plays a critical role in the management of non-Hodgkin lymphomas involving the testis when carefully planned and administered [1].

Unfortunately, there is no standard testicular irradiation workflow, and many oncologists rely on case reports to deliver adequate treatment, especially in terms of positioning, target volume delineation, and dose prescription [2].

In order to review the different techniques used to irradiate testicular cancer, we have used for this article a case of unilateral localized primary testicular cancer (PTL) in a 78-year-old patient for illustration purposes.

## Case presentation

We report the case of a 78-year-old man, a chronic smoker, treated for high blood pressure with monotherapy (amlodipine). The patient presented with an acute swollen right testicular sac, occurring outside of any trauma, fever, night sweats, or weight loss.

The scrotal ultrasonography showed an increase in the right testicular size, measuring 52x50x49 mm with a volume of 66 cc. It was heterogenic and hyper-vascularized, suggesting an infectious etiology. Testicular exploration was performed under spinal anesthesia. A horizontal right semi-scrotal incision was made and the scrotal envelops were opened to expose the right abscessed testicle, which was removed.

The histopathology of the orchiectomy specimen reported an undifferentiated tumoral proliferation in the whole testicle, with hemorrhagic foci. The tumor measured 10x7,5x5 cm and was made of small and medium-sized cells, invading the spermatic cord, with safe margins.

Immunochemistry showed background reactive T cells positive for CD3 (cluster of differentiation 3), a protein complex, and T cell co-receptor that is involved in activating both the cytotoxic T cell (CD8+ naive T cells) and T helper cells (CD4+ naive T cells)) and CD5, diffuse strong positive for CD20 in the neoplastic lymphoid cells, diffuse nuclear positive for BCl 6 (B-cell lymphoma 6, a protein that is encoded by the BCL6 gene) and MUM1 (multiple myeloma oncogene1) in the neoplastic lymphoid cells. A positive Ki67 index of 85% was also reported. All these data confirmed a diffuse large B-cell lymphoma (DLBC) of a non-germinal center type.

Postoperative serum tumor markers (alpha-fetoprotein, β-human chorionic gonadotropin), albumin, and lactate dehydrogenase levels were normal. VIH 1 and 2 serologies tested negative.

Due to a delay in obtaining an initial appointment for a fluorodeoxyglucose (FDG)-positron emission tomography (PET) scan, an initial evaluation workup was performed by a whole-body computed tomography, an osteo-medular biopsy, and a lumbar puncture. They respectively all ruled out any distant visceral, bone marrow, or cerebrospinal metastasis.

After discussing this patient’s case of a stage IA testis diffuse large B-cell lymphoma in a hematology tumor board, the decision was to administrate chemotherapy followed by radiation therapy for this primary testicular lymphoma.

The patient was started on R-mini CHOP protocol: a reduced dose chemotherapy with the following molecules: cyclophosphamide (400 mg per m2) day 1; doxorubicin (25 mg per m2) day 1; vincristine 1 mg total dose day 1 and prednisolone (40 mg per m2) by oral route from day 1 to day 5; plus rituximab (375 mg per m2) day 1; every 21 days for six cycles. He then received two cycles of reduced dose intrathecal methotrexate (1,5g per m2) triweekly.

Technical report

**Figure 1:**
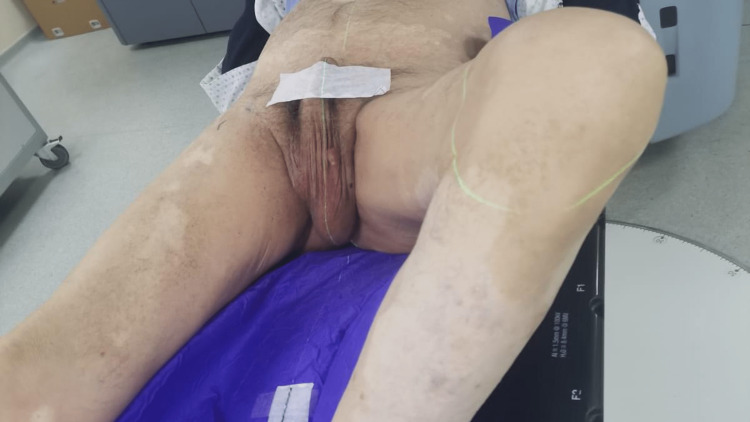
First simulation CT of the patient

The patient received radiation therapy. The challenge was to obtain a comfortable, reproducible, and efficient treatment position. The dosimetric computed tomography (CT) was performed in the following position: the patient was lying on his back with a hip anteflexion. To expose the controlateral testis to irradiation, the patient’s thighs were spread, the left knee bent, and rotated to the left and the right one extended. His hands were placed on his chest (Figure 1).

We used a total body restraint system (Vac-lok) for immobilization and reproducibility purposes.

In order to protect the penis and for reproducibility matters, it was fixated against the pubic wall, and the urethral orifice position was tattooed on the patient’s skin. We did not inject an iodinated contrast agent, as the scrotum was well visualized without it.

**Figure 2:**
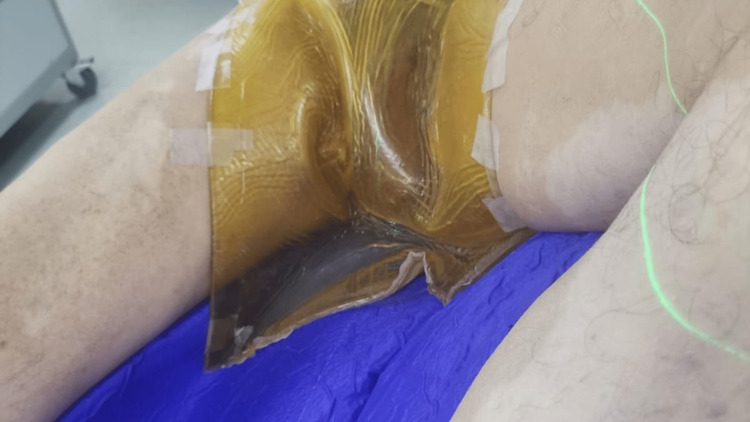
Second simulation CT with the bolus on the region of interest

The CT images were obtained by thin sections of 2.5 mm and exported to the treatment planning system. A first treatment planning and dosimetry were performed but the dose was not optimal, as it did not cover the superficial layers of the scrotum. We then performed a second simulation CT scan using a bolus fixed on the patient’s skin, as shown in Figure 2.

**Figure 3:**
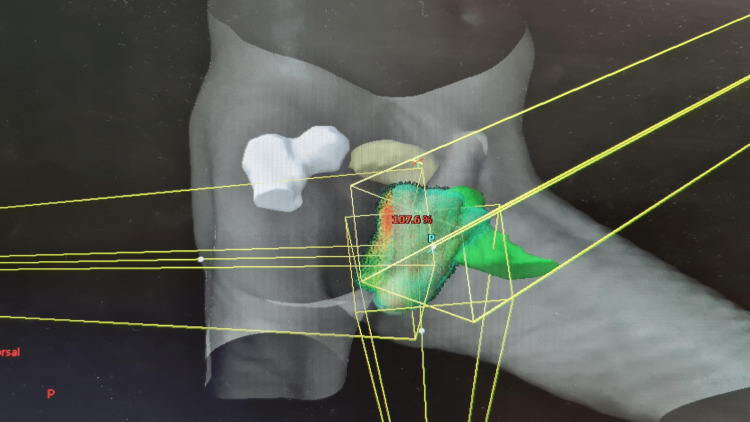
Photon beam arrangements for conformal radiotherapy

For this postoperative case, there was no gross tumoral volume (GTV). The entire scrotum was delineated as the clinical target volume (CTV), according to 2018 guidelines of the International Lymphoma Radiation Oncology Group (ILROG). We added a 1 cm margin around the CTV to obtain the planning target volume (PTV). Rectum, anal canal, bladder, and femoral heads were delineated as organs at risk (Figure 3).

**Figure 4:**
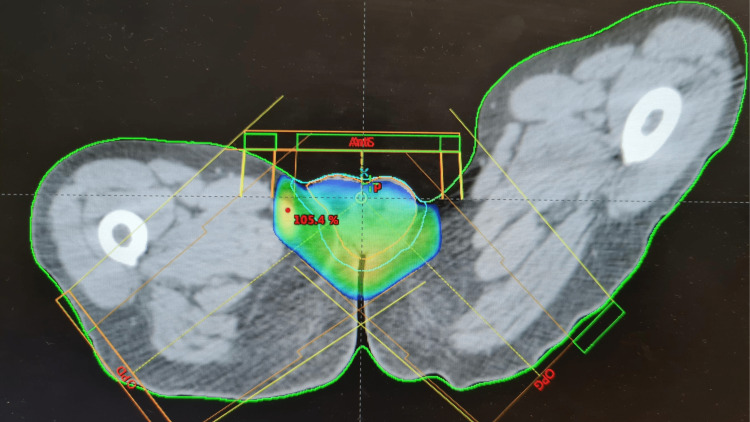
Target volumes and dosimetric outcomes

The treatment planning by a three-dimensional conformational technique was performed with one anterior beam and two posterior oblique beams with wedge filters. Six and 18 MV photons were applied, as shown in Figure 4.

The prescribed dose was 30 Gray (Gy) with 2 Gy per fraction and five sessions per week for 15 sessions total. The treatment was well-tolerated, and the patient presented a first-degree acute radiodermatitis, managed by symptomatic treatment, with good evolution.

The whole-body 18-fluorodeoxyglucose positron emission tomography-computed tomography (18-FDG-PET-CT) performed three months after irradiation confirmed total remission.

## Discussion

Testicular radiation therapy

Testicular radiotherapy, which is indicated for non-Hodgkin lymphomas, is applied to the involved testis (if not resected) and to the remaining testis and scrotum due to the high risk of the contralateral testis relapse [1-2].

In terms of outcomes, overall survival and scrotal control were significantly improved by radiotherapy. The results of several retrospective studies indicate that adjuvant irradiation to the scrotum is associated with better survival. Zucca et al. showed the median OS in patients receiving radiation therapy was 5.9 years versus 2.0 years in patients not being irradiated [3-5]. Without scrotal irradiation, scrotal relapse of up to 35% was observed, compared with 0%-10% in patients after scrotal irradiation.

Technically, the challenge in scrotal irradiation is maintaining a reproducible patient positioning. The scrotum is a very mobile sac that changes in size due to temperature, emotions, or others. the patient is positioned supine in a frog-leg situation, the penis is lifted and taped to the abdominal wall, and the scrotum is supported and immobilized with a bolus under and around it as described above. It is ensured that patient positioning should be assisted by the construction of patient-specific molds, but thankfully, our patient’s irradiation was optimal, as his position seemed to assure the correct level of reproducibility, based on clinical and surface imaging verification [5].

In most studies, the target volume for local irradiation was defined as the scrotum. Some physicians also included nodal irradiation [6-7].

The prescription doses mentioned in various studies ranged from 18-56 Gy, favoring a prescribed dose of at least 30 Gy involving the scrotum since it’s associated with improved contralateral testis control and minimal toxicity [8-9].

Treatment plan optimization and better sparing of the organs at risk (OAR) is possible by putting the patients in a wide-leg position. When a single photon beam is used, an option might be to put some attenuation material behind the scrotum to reduce the anal dose. Our patient’s position allows us to expose the scrotum to irradiation. However, the anal canal’s dose cannot be lowered in this position and no additional protective material was available for use for our patient [10-11].

The treatment is usually delivered by conformal radiotherapy technique (3D). More recent techniques could be used, but regarding the long-term survival of these patients, it is preferred to stick to a 3D technique to avoid any potential increased risk of secondary cancer [12].

While some institutes may still use outdated techniques, such as irradiating the whole scrotum with a single electron beam without a planning computed tomography (CT) scan, modern precision radiotherapy allows for targeted treatment delivery to the testis only. This approach ensures that the treatment is localized to the intended target while minimizing potential side effects to surrounding healthy tissues. With the prognosis of well-managed testicular lymphoma being generally good, it is crucial to prioritize the use of quality radiotherapy techniques to achieve optimal treatment outcomes [13-14].

As for side effects, infertility, reflecting the insufficient exocrine function of the testis, is expected in all men after an irradiation dose of 30 Gy [15]. The potential castration effect of radiotherapy was not a concern in our patient's case, as he had previously undergone an orchiectomy and received a chemotherapy protocol that could potentially impact fertility [16].

## Conclusions

Testicular radiation therapy can be an essential component of the overall treatment of some testicular neoplasms. However, due to the rarity of this disease, there is no standard workflow for this specific irradiation site. Therefore, oncologists often rely on case reports to deliver adequate treatment, especially in terms of positioning, target volume delineation, and dose prescription. In the case presented, we demonstrated the challenges of delivering radiation therapy to a patient with primary testicular lymphoma. The technical report highlights the importance of obtaining a comfortable, reproducible, and efficient treatment position and using immobilization techniques for the optimal delivery of radiation. The use of a bolus to cover the superficial layers of the scrotum is also essential to ensure adequate dose coverage. In summary, improving the standardization of testicular irradiation workflows and sharing experiences through case reports can lead to better outcomes for patients with testicular cancer.
